# Ferroptosis in Cancer Progression: Role of Noncoding RNAs

**DOI:** 10.7150/ijbs.66917

**Published:** 2022-02-14

**Authors:** Ying-Bing Zuo, Yin-Feng Zhang, Rui Zhang, Jia-Wei Tian, Xiao-Bing Lv, Rong Li, Shu-Ping Li, Meng-Die Cheng, Jing Shan, Zheng Zhao, Hui Xin

**Affiliations:** 1 Department of Cardiology, The Affiliated Hospital of Qingdao University, Qingdao University, Qingdao 266000, Shandong, P.R. China.; 2 Institute for Translational Medicine, The Affiliated Hospital of Qingdao University, College of Medicine, Qingdao University, Deng Zhou Road 38, Qingdao 266021, Shandong, P.R. China.; 3 Department of Emergency Internal Medicine, The Affiliated Hospital of Qingdao University, Qingdao 266000, Shandong, P.R. China.; 4 Department of Cardiology, The Affiliated Qingdao Third People's Hospital of Qingdao University, Qingdao University, Qingdao 266041, Shandong, P.R. China.; 5 Key Laboratory of Environmental Factors and Chronic Disease Control, Yinchuan 750004, Ningxia, P.R. China.; 6 School of Public Health and Management, Ningxia Medical University, Yinchuan 750004, Ningxia, P.R. China.

**Keywords:** Ferroptosis, microRNAs, long noncoding RNAs, circular RNAs, Cell death

## Abstract

Ferroptosis is a novel form of programmed cell death, and it is characterized by iron-dependent oxidative damage, lipid peroxidation and reactive oxygen species accumulation. Notable studies have revealed that ferroptosis plays vital roles in tumor occurrence and that abundant ferroptosis in cells can inhibit tumor progression. Recently, some noncoding RNAs (ncRNAs), particularly microRNAs, long noncoding RNAs, and circular RNAs, have been shown to be involved in biological processes of ferroptosis, thus affecting cancer growth. However, the definite regulatory mechanism of this phenomenon is still unclear. To clarify this issue, increasing studies have focused on the regulatory roles of ncRNAs in the initiation and development of ferroptosis and the role of ferroptosis in progression of various cancers, such as lung, liver, and breast cancers. In this review, we systematically summarized the relationship between ferroptosis-associated ncRNAs and cancer progression. Moreover, additional evidence is needed to identify the role of ferroptosis-related ncRNAs in cancer progression. This review will help us to understand the roles of ncRNAs in ferroptosis and cancer progression and may provide new ideas for exploring novel diagnostic and therapeutic biomarkers for cancer in the future.

## 1. Introduction

### 1.1 Mechanism of ferroptosis

Programmed cell death (PCD) is important for the balance between the progression of diseases and human health 1. Ferroptosis, a novel coined form of PCD discovered in 2012 2, is different from apoptosis, necroptosis, pyroptosis and autophagy 3. Many studies have revealed that ferroptosis is a specific oxidative and iron-dependent form of PCD caused by abnormal iron metabolism and lethal lipid peroxidation 4,5. Moreover, some studies have demonstrated that autophagy plays a crucial role in ferroptosis, especially autophagic degradation of ferroptosis-related proteins, such as ferritinophagy, lipophagy, clockophagy and chaperone-mediated autophagy 6,7. Recently, an increasing number of studies have focused on the role of ferroptosis in various diseases 8,9, especially liver, lung, and gastrointestinal cancers 10. By exploring the molecular mechanism related to the regulation of ferroptosis more deeply, the relationship between ferroptosis and cancer progression will be better understood.

**Fig 1:**
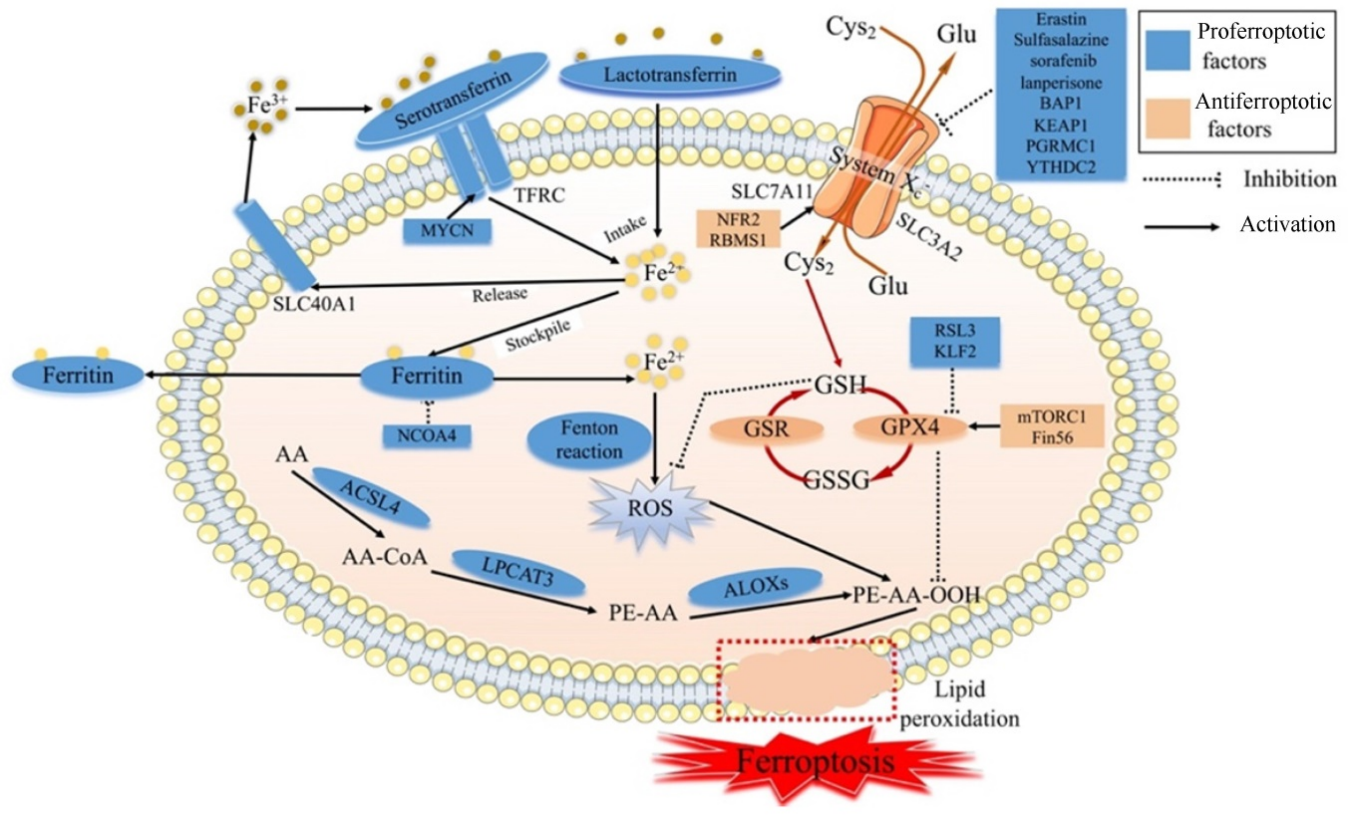
** The molecular mechanism of ferroptosis.** Cys_2_: Cysteine; Glu: Glutamate; SLC7A11: Solute carrier family 7 membrane 11; SLC3A2: Solute carrier family 3 membrane 2; SLC40A1: Solute carrier family 40 membrane 1; TFRC: Transferrin receptor; GSH: Reduced glutathione; GPX4: Glutathione peroxidase 4; GSR: Glutathione reductase; GSSG: Oxidized Glutathione; ROS: Reactive oxygen species; AA: Arachidonic acid; ACSL4: Acyl-CoA synthetase long-chain family member 4; LPCAT3: lysophophatidylcholne acyltransferase 3; ALOXs: Lipoxygenases

Ferroptosis is regulated by specific signal transduction pathways through iron accumulation, lipid peroxidation and cellular membrane destruction, and ferroptosis can be modulated by drugs or genetic interventions 11 (Fig. 1). The main mechanism of ferroptosis involves regulating homeostasis between oxidative and antioxidant systems 12.

#### 1.1.1 Iron in ferroptosis

Iron accumulation plays a critical role in producing ROS via the Fenton reaction and enzyme activity in terms of lipid peroxidation. Although iron is essential in physiological processes, excessive iron is pernicious and can trigger ferroptosis. Ferroptosis is strictly regulated by modulators related to iron metabolism processes, such as iron intake, stockpile, usage, and release 13. Serotransferrin- or lactotransferrin-associated iron intake promotes ferroptosis through the transferrin receptor (TFRC) 14,15. Furthermore, oncogenic MYCN could induce iron accumulation by increasing the expression of TFRC 16. The cargo receptor NCOA4 could activate autophagy to degrade ferritin, a process called ferritinophagy, leading to the promotion of ferroptosis 17. In contrast, solute carrier family 40 membrane 1 (SLC40A1)-associated iron release inhibits ferroptosis 18. Along with the degradation of ferritin, the level of intracellular iron is high, leading to ferroptosis 19, whereas ferritin efflux inhibits ferroptosis. Evidence revealed that the transcription factor BACH1 could reduce iron accumulation by upregulating the translation of ferritin genes (Fth1 and Flt1) and the ferroportin gene (SLC40A1) and inhibiting ferroptosis 20. Some mitochondrial proteins associated with the usage of iron negatively regulate ferroptosis, such as NFS1 21, ISCU^22^, CISD1 23 and CISD2 24. Moreover, the iron accumulation could be regulated by some signal transduction pathways. Nuclear protein 1 (NUPR1) , a transcriptional regulator, blocks cell ferroptosis and decreases iron accumulation by increasing the production of the iron transporter LCN2 25. Recent studies have revealed that iron chelators and antioxidants can inhibit ferroptosis 26-28.

#### 1.1.2 Lipid peroxidation in ferroptosis

Lipid peroxidation leading to cell membrane destruction is the central intermediate link of ferroptosis. The molecular mechanism of lipid peroxidation is that the inhibition of system Xc^-^ or glutathione peroxidase 4 (GPX4) leads to decreased production of reduced GSH 14. The glutamate (Glu)/cysteine (Cys_2_) antiporter of system Xc^-^ is composed of solute carrier family 3 membrane 2 (SLC3A2) and solute carrier family 7 membrane 11 (SLC7A11) and can import extracellular Cys_2_ into cells and exchange intracellular Glu. Moveover, some evidence has revealed that the cargo receptor SQSTM1/p62 can induce the autophagic degradation of ARNTL, known as clockophagy, and promote lipid peroxidation and ferroptosis via the EGLN2/HIF1A pathway 29,30.

##### Inhibiting system Xc^-^

System Xc^-^, which involves GSH, is one of the main antioxidant defenses in our body. When system Xc^-^ is inhibited by erastin, Cys_2_ cannot influx cells, resulting in a decrease in the amount of Cys_2_. Due to the important role of Cys_2_ in the process of GSH biosynthesis, Cys_2_ deficiency can reduce the level of GSH. Then, the exhaustion of GSH can reduce the expression and activity of GPX4 31,32. Meanwhile, GPX4 can catalyze GSH to GSSG, and then toxic peroxides can be reduced to nontoxic hydroxyl compounds to protect the structure and function of the cell membrane from interference and destruction by peroxides 33. Along with the destruction of the redox balance in cells, the accumulation of ROS can induce cell membrane breakage and cell death 34. The imbalance of system Xc^-^ is one of the main biochemical features of ferroptosis, and the regulation of ferroptosis is the primary way to control its occurrence.

Several studies uncovered that ferroptosis could be induced by inhibiting system Xc^-^ via certain compounds, such as erastin, sulfasalazine 4, sorafenib, and lanperisone 35. Moveover, studies have identified that some genes could regulate the catalytic subunit of system Xc^-^. These genes regulate the transcription or translation of subunits of system Xc^-^, such as SLC7A11 or SLC3A2, affecting the biological processes of the ferroptosis. The tumor suppressor BRCA1-related protein1 (BAP1) could inhibit the SLC7A11 expression and then lead to elevated lipid peroxidation and ferroptosis 36. In some cancers, Kelch-like ECH-associated protein 1 (KEAP1) depresses the translation of SLC7A11 and reduces the exchange of Glu/Cys_2_, otherwise NF E2-related factor 2 (NRF2) plays the opposite role in SLC7A11 37. Hence, the Nrf2/Keap1 pathway promotes the translation of SLC7A11, leading to diminished ferroptosis 38. In lung cancer, the RNA-binding protein RBMS1 could promote the levels of SLC7A11 and increase the production of GSH, resulting in the inhibition of ferroptosis in cancer cells 39. In terms of the relationship between autophagy and lipid peroxidation, progesterone receptor membrane component 1 (PGRMC1) suppresses SLC7A11 via autophagic degradation of lipids, known as lipophagy, and induces ferroptosis in paclitaxel‐tolerant persister cancer cells 40. P53, a well-known tumor suppressor gene, can inhibit the expression of SLC7A11, leading to lipid peroxidation and ferroptosis 41. YTHDC2, an m^6^A reader identified in 2017, suppresses the expression of SLC3A2 by inhibiting HOXA13, a transcription factor of SLC3A2 expression, to trigger system Xc^-^ -dependent ferroptosis 42.

##### Inhibiting GPX4

GPX4, another main antioxidant defense, can directly reduce cell membrane phospholipid hydroperoxide to hydroxyphospholipid, taking advantage of GSH as a substrate, resulting in the suppression of ferroptosis in cancer cells 43. The inhibition of GPX4 by RSL3 induces ferroptosis 44. Recently, many studies have revealed that some factors regulate the generation process of GPX4. Boyi Gan and his team discovered that rapamycin complex 1 (mTORC1), a foremost regulator of cell growth and metabolism, increased the production of GPX4 protein and reduced the production of lipid peroxidation of the cell membrane 45-47. Moreover, Fin56, a ferroptosis inducer, synergized with Torin 2, promoting GPX4 translation and triggering ferroptosis in bladder cancer cells 48. In other types of cancer, KLF2 reduces the transcriptional repression of GPX4 to prevent the lipid peroxidation and inhibit ferroptosis in clear cell renal cell carcinoma 49. Interestingly, erastin activation of ferroptosis increased the production of lysosome-associated membrane protein 2a and induced chaperone-mediated autophagy, which in turn increased the degradation of GPX4 resulting in reduced ferroptosis 50.

Genetic depletion of GPX4 causes lipid peroxidation and then induces ferroptosis in cancer cells or tissues 51. In the process of lipid metabolism, arachidonic acid (AA) can produce AA-CoA through acyl-CoA synthetase long-chain family member 4 (ACSL4), and AA-CoA is esterified by lysophophatidylcholine acyltransferase 3 (LPCAT3) to produce phosphatidyl-(PE)-AA 52. PE-AA is oxidized to PE-AA-OOH by lipoxygenases (LOXs), leading to degradation of the cell membrane 53. Cytotoxic PE-AA-OOH is usually reduced to noncytotoxic PE-AA-OH, protecting cells from oxidative damage via GPX4. However, when GPX4 is deficient or inactivated, PE-AA-OOH cannot be reduced and then induces ferroptosis 53. Overall, GPX4 systems are also crucial for the occurrence of ferroptosis.

#### 1.1.3 ROS in ferroptosis

In the process of lipid peroxidation, the lethal accumulation of lipid ROS can destroy the cell membrane, leading to ferroptosis 54. ROS are produced in two main ways: the Fenton reaction with Fe^2+^ and lipid peroxidation. When the two antioxidant defenses, GSH and GPX4 systems, are impaired, the accumulation of toxic ROS will occur and then induce cell death. Some studies have revealed that erastin causes the production of ROS in some cell lines 4,55. Cells treated with RSL3 revealed elevated lipid ROS during ferroptosis in the absence of GSH depletion. With prolonged erastin and RSL3 treatment times, ROS can begin to accumulate and induce cancer cell ferroptosis 4 Furthermore, ROS are generated from the TCA cycle of mitochondrial metabolism.

### 1.2 Inhibition of cancer progression by ferroptosis

Many studies have indicated that ferroptosis plays a crucial role in the regulation of the pathological process of cancer 56,57. Some studies have revealed that superabundant ferroptosis of cancer cells can inhibit tumor progression. Several anticancer drugs could inhibit ferroptosis-related molecules and channels to induce ferroptosis in cancer cells, such as GPX4 and system Xc^-^, and then inhibit cancer growth 5,58. It was found that the ferroptosis inducer erastin could increase the chemotherapeutic effect of some chemotherapeutics, such as cisplatin 59, cytosine arabinoside and doxorubicin 60, by inducing ferroptosis. Similarly, inactivation of dihydroorotate dehydrogenase led to a large amount of mitochondrial lipid peroxidation and induced ferroptosis in cancer cells 61. Moreover, radiotherapy could cause cancer cells to produce lipid ROS and result in the lethal accumulation of lipid peroxides to induce ferroptosis 62. Hence, the induction of ferroptosis may become a promising strategy to treat cancer. Next, we will discuss the relationship between ncRNAs and ferroptosis.

### 1.3 Function of ncRNAs in ferroptosis

**Fig 2:**
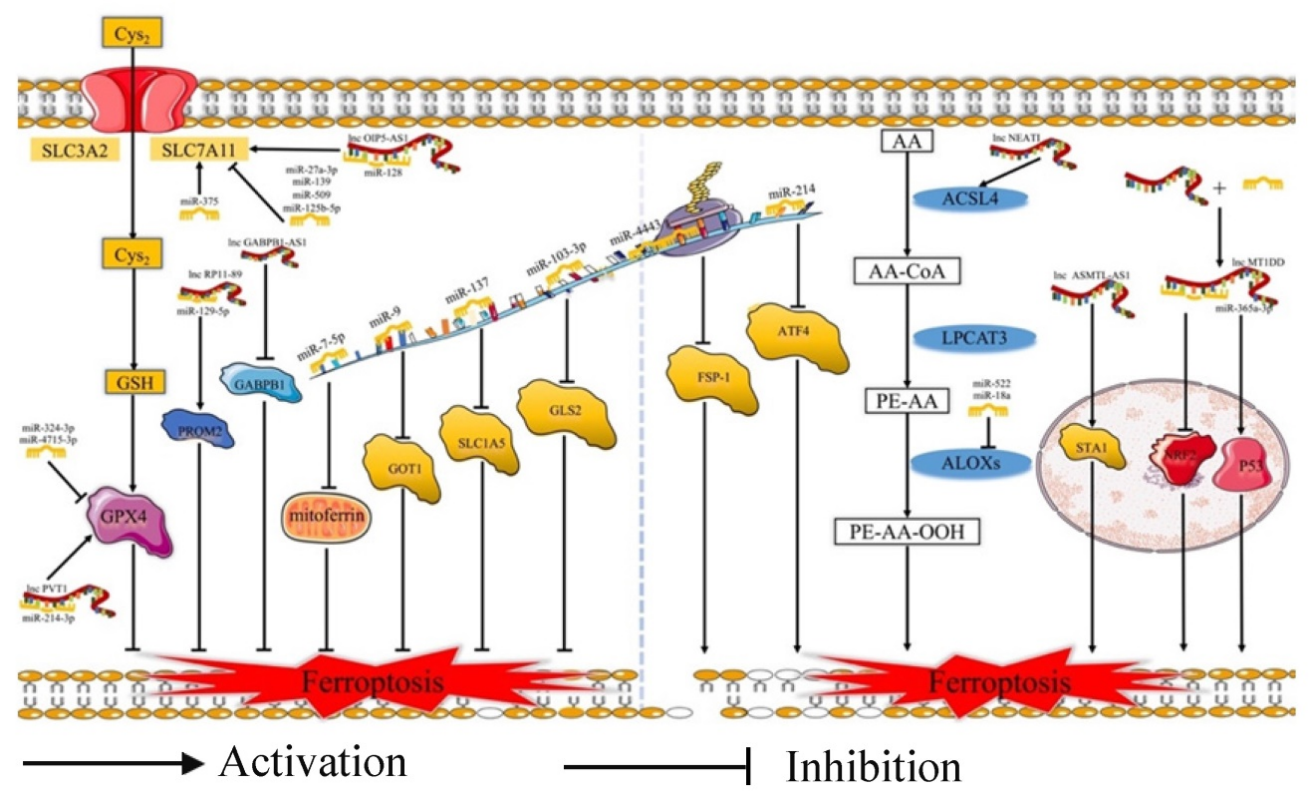
** MiRNAs and lncRNAs play regulatory role in ferroptosis.** Cys_2_: Cysteine; GSH: Reduced glutathione; SLC3A2: Solute carrier family 3 membrane 2; SLC7A11: Solute carrier family 7 membrane 11; GPX4: Glutathione peroxidase 4; GOT1: Glutamic-oxaloacetic transaminase 1; SLC1A5: Solute carrier family 1 membrane 5; GLS2: Glutaminase 2; FSP-1: Ferroptosis suppressor protein 1; ATF4: Activating transcription factor 4; AA: Arachidonic acid; ACSL4: Acyl-CoA synthetase long-chain family member 4; LPCAT3: lysophophatidylcholne acyltransferase 3; ALOXs: Lipoxygenases

**Fig 3:**
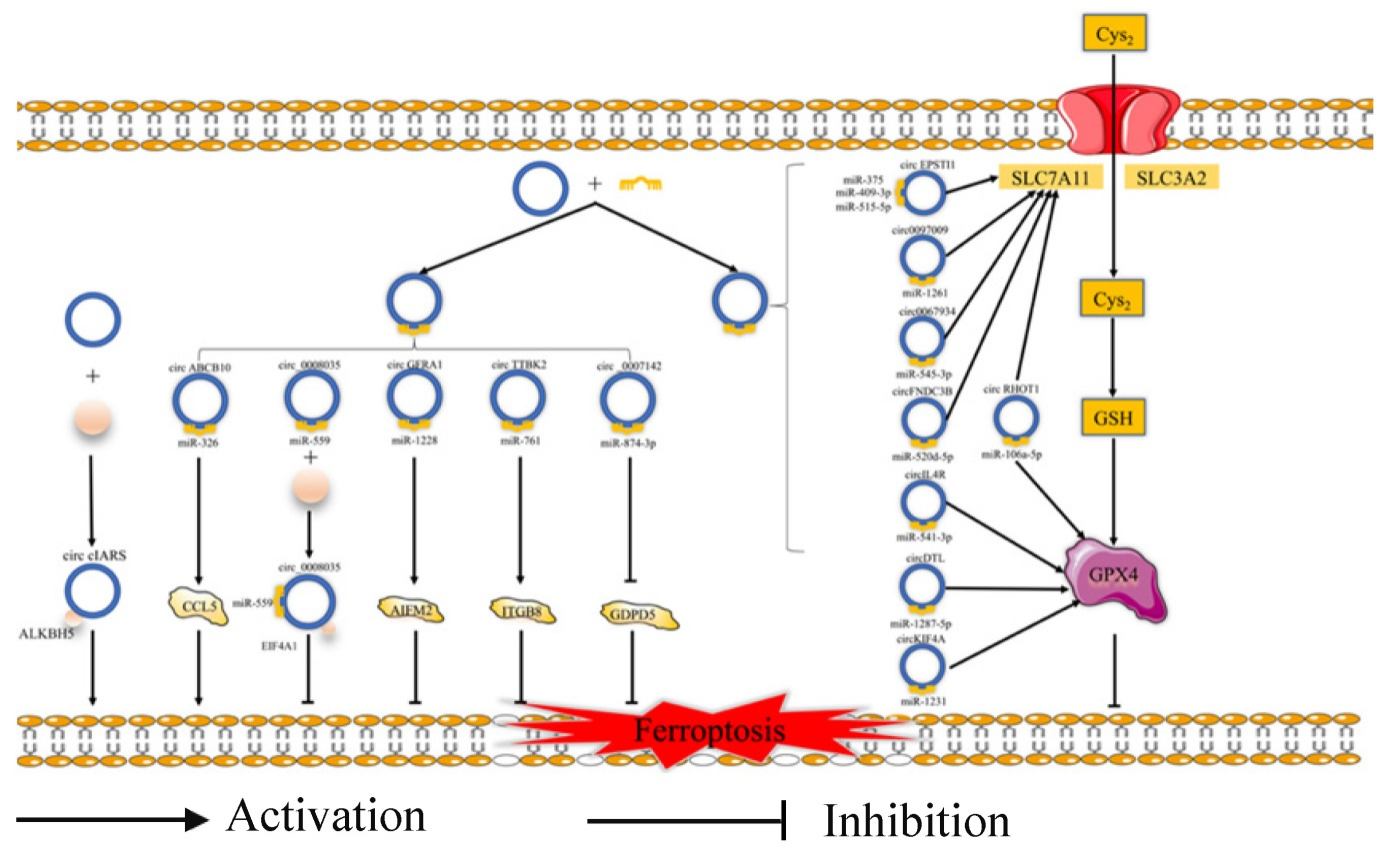
** The regulatory role of circRNAs in ferroptosis.** Cys_2_: Cysteine; GSH: Reduced glutathione; SLC3A2: Solute carrier family 3 membrane 2; SLC7A11: Solute carrier family 7 membrane 11; GPX4: Glutathione peroxidase 4; ALKBH5: Alk B homologue 5; CCL5: Chemokine (C-C motif) ligand 5; EIF4A1: Eukaryotic Translation Initiation Factor 4A1; ITGB8: Integrin beta 8; GDPD5: Glycerophosphodiester phosphodiesterase domain containing 5.

Ferroptosis is related to the prognosis of many types of cancer, but we know little of the mechanism of the ferroptosis in cancer, especially regarding the role of ferroptosis-related ncRNAs in cancer. Ferroptosis is tightly related to noncoding RNAs (ncRNAs) and cancer 63. NcRNAs, including microRNAs (miRNAs), long noncoding RNAs (lncRNAs) (Fig. 2), and circular RNAs (circRNAs) (Fig. 3), are involved in the underlying regulatory mechanism of ferroptosis, including mitochondrial-related proteins, iron metabolism, glutathione metabolism, and lipid peroxidation 64,65.

In terms of regulating ferroptosis-related genes, some studies have demonstrated that many ncRNAs play vital roles in regulating the expression of the ferroptosis-related genes 66. Some ncRNAs regulate ferroptosis in cancer cells by affecting the protein level of ferroptosis-associated genes, such as FSP1 67, EIF4A1 68, GABPB1 69, GDPD5 70, and CCL5 71. A number of ncRNAs could affect both the mRNA and protein levels of ferroptosis-associated genes, such as NRF2 72, STAT3 73, ATF4 74, AURKA 75, and ITGB8 76. What's more, several miRNAs could participate at the mRNA or protein level via m6A modification or epigenetic regulation of these genes, such as FSP1 67 and AURKA 75. Some circRNAs could regulate these genes by sponging miRNAs at the mRNA and protein levels. Moreover, lncRNAs could regulate the expression of ferroptosis-related proteins by affecting p53 at the transcriptional level. For example, lncRNA P53RRA could promote the recruitment of p53 by interacting with G3BP1 and then regulate the function of ferroptosis-associated metabolic genes 77. Moreover, some ncRNAs could induce ferroptosis by regulating iron metabolism. Some studies have revealed that many ncRNAs increase the content of cellular iron 72,77-79. Apart from this, several ncRNAs could decrease the accumulation of iron 80-82. Moreover, miR-7-5p plays a vital role in downregulating mitoferrin, reducing Fe^2+^ 83, and inhibiting the ferroptosis. In addition, many ncRNAs could affect ferroptosis via ROS metabolism by the Fenton reaction 69,73,79,80. However, future studies need to pay more attention to the molecular mechanism of the relationship between ferroptosis-related ncRNAs and iron or ROS metabolism.

Regarding lipid peroxidation, ncRNAs regulate the subunits of system Xc^-^ and GPX4, and several ncRNAs play important roles in the process of lipid metabolism. Many ncRNAs regulated the protein level of SLC7A11 78,84-87. Moreover, some circRNAs regulated SLC7A11 by sponging miRNAs. Furthermore, several studies revealed that ncRNAs could increase the levels of SLC7A11 by promoting the recruitment of LSH to the promoter of SLC7A11 79. In the aspect of GPX4, many studies have demonstrated that ncRNAs focus on regulating the protein production of GPX4 88-92. A few studies have revealed that circRNAs can upregulate the levels of GPX4 mRNA 91. Several studies have demonstrated that lncRNAs and circRNAs can regulate the GPX4 by interacting with miRNAs. For instance, lncRNA PVT1 induced the expression of GPX4 by inhibiting the translation of miR-214-3p 90. In addition, has_circ_0048179 increased the level of GPX4 by sponging miR-188-3p 93. Regarding the uptake of Gln, miRNAs could regulate the expression of the Glu metabolism-related proteins and affect the ferroptosis of cells. For example, miR-103a-3p inhibited the expression of glutaminase 2 (GLS2) and suppressed the glutamine transformation to glutamate 94. miR-9 could inhibit the mRNA and protein levels of glutamic-oxaloacetic transaminase 1 (GOT1) and inhibit Glu metabolism 95. In addition, miR-137 inhibited glutaminolysis by suppressing the glutamine transporter solute carrier family 1 membrane 5 (SLC1A5) 81. Moreover, several studies have revealed that ncRNAs can induce lipid peroxidation by regulating the translation of the fundamental enzymes for the biosynthesis of unsaturated phospholipids, such as ACSL4 96,97, ALOX15 80, and ALOXE3 98. In summary, ncRNAs play crucial roles in many biological processes of ferroptosis occurrence and development.

Recently, an increasing amount of evidence has demonstrated that ncRNAs play an important regulatory role in cancer progression via the ferroptosis pathway 3,64 and might become new diagnostic markers or therapeutic targets of cancers. Hence, this review focuses on summarizing the regulatory role of ncRNAs in cancer progression via ferroptosis of cancer cells (Table 1). Moreover, there may be some obstacles to hindering the exploration of ferroptosis-related ncRNAs in cancer therapy or diagnosis. We believe that a deep understanding of the interactions between ncRNAs and ferroptosis may be conducive to solving these obstacles and improving the strategy of cancer therapy or diagnosis.

## 2. Role of ncRNAs in cancer progression via the ferroptosis pathway

### 2.1 Lung cancer

Lung cancer is the leading cause of cancer-associated deaths, with approximately 1.8 million deaths (18%), and it is the most common cancer in men worldwide, with an estimated 1.44 million new cases each year (14.3%) 99. Lung cancer is divided into small cell lung cancer (SCLC) and non-small-cell lung cancer (NSCLC). NSCLC, including lung adenocarcinoma (ADC) and squamous cell carcinoma (SCC), is the most frequently occurring cancer among lung cancers 100. Although there are many therapeutic strategies for lung cancer therapy, such as surgical sectioning, chemotherapy, and radiotherapy, there is still a lack of a definite understanding of the pathogenesis of lung cancer. Thus, identifying novel diagnostic markers and therapeutic strategies to inhibit the progression of lung cancer is a great challenge. However, second-generation sequencing technology has been widely used to provide a very effective way to study disease-related genes and ncRNAs in recent years 101,102. To a certain extent, this technology will help us to predict ferroptosis-related ncRNAs.

Recently, miRNAs have attracted substantial attention in lung cancer. Several miRNAs play important roles in chemotherapeutic resistance via ferroptosis. For example, exosomal miR-4443 could increase cisplatin resistance to reduce the therapeutic effect of NSCLC via METTL3/FSP1-induced ferroptosis 67. Moreover, Shi-hua Deng 88 demonstrated that miR-324-3p could reduce cisplatin resistance by inducing GPX-mediated ferroptosis in ADC cells. Research revealed that miR-27a-3p could induce ferroptosis by suppressing the expression of SLC7A11 in NSCLC 86. The regulatory role of miRNAs in ferroptosis might contribute to an in-depth understanding of the mechanism of chemoresistance in NSCLC and uncover potential therapeutic methods for improving chemotherapeutic resistance in NSCLC.

Furthermore, lncRNAs also play relevant roles in lung cancer progression. In 2018, Chao Mao 77 demonstrated that the lncRNA P53RRA could directly interact with the functional domain of G3BP1, resulting in abnormal accumulation of p53 in the nucleus to induce cell arrest and ferroptosis and then inhibit lung cancer progression. In contrast, it was reported that a novel lncRNA, LINC00336, which sponges miR-6825, served as a competing endogenous RNA (ceRNA) and promoted lung cancer proliferation by inhibiting ferroptosis 82. In terms of NSCLC, some studies revealed that several ferroptosis-associated lncRNAs could inhibit tumor deterioration via ferroptosis. For instance, sponging lncRNA miR-503HG with miR-1273c may inhibit NSCLC progression via ferroptosis 103. Similarly, Hong-xia Wu 96 showed that lncRNA NEAT1 upregulated the expression of ACSL4 associated with ferroptosis and inhibited the worsening of NSCLC. Another study uncovered that lncRNA MT1DP enclosed by folate-modified liposome nanoparticles via miR-365a-3p/NRF2 could improve the sensitivity to ferroptosis and might become a new therapeutic method for NSCLCs 72. This research revealed that nanoparticles could be used to treat cancers by interacting with ncRNAs. Regarding ADC, lncRNA ASMTL-AS1 could inhibit cancer progression and promote ferroptosis by stabilizing SAT1 via recruiting U2AF2 104. In addition, several ferroptosis- and iron metabolism-related lncRNAs have been identified as prognostic biomarkers of ADC 105,106. Several studies have focused on the role of circRNAs in NSCLC 107,108. For example, circDTL reduced the ferroptosis of cancer cells via the miR-1287-5p/GPX4 pathway 108.

However, more evidence is needed to explore the regulatory mechanism of ncRNAs in lung cancer via the ferroptosis pathway, and the regulatory roles of other ncRNAs in ferroptosis in lung cancers remain to be discovered.

### 2.2 Gastrointestinal cancer

Gastrointestinal cancer is also a serious leading cause threatening the health of humans worldwide, and its death rate is estimated to be 1.69 million (17.1%) 99. Its type is divided into upper gastrointestinal cancers (UGCs), including gastric cancer (GC), and lower gastrointestinal tumors, including colorectal cancer (CRC). Due to the lack of adequate knowledge of early symptoms of gastrointestinal cancer, most patients are apt to miss the optimum therapeutic period in the early stage. Thus, the disease has a huge adverse impact on family and society. Therefore, it is essential to deeply explore the pathological mechanism of gastrointestinal cancer and uncover novel diagnostic markers or therapeutic methods to diagnose or treat it in the early stage.

To explore the molecular mechanism of UGC, Ahmed Gomma 75 revealed that overexpression of miR-4715-3p could reduce Aurora kinase A levels, leading to G2/M delay of cells, and inhibiting GPX4 resulted in the ferroptosis of UGC cells. Moreover, miR-139 could inhibit SLC7A11-mediated ferroptosis via the PI3K/Akt signaling pathway and suppress the prolifetation of pancreatic carcinoma 109. miR-375 could reduce the regeneration ability of GC cells by triggering SLC7A11-mediated ferroptosis 110. In terms of chemotherapeutic resistance in GC, some evidence has revealed that miRNAs play an important role in chemotherapeutic resistance 80,111. For example, exo-miR-522 secreted by cancer-associated fibroblasts interacted with ALOX15 to promote acquired chemotherapeutic resistance in GC by inhibiting ferroptosis in GC cells 80. Moreover, Ying Niu 94 revealed that physcion 8-O-β-glucopyranoside, a chemical component contained in Rumex japonicas Houtt, could induce ferroptosis and suppress the proliferation and metastasis of GC cells via the miR-103a-3p/GLS2 pathway and inhibit the growth and metastasis of GC.

Not only do miRNAs play regulatory roles in the proliferation and metastasis of gastrointestinal cancer, but lncRNAs also have important effects on improving gastrointestinal cancer progression 112,113. For example, Hua-jun Cai 114 investigated and constructed seven ferroptosis-related lncRNA signature by a Cox regression model to predict the survival of colon adenocarcinoma patients. An increasing number of ferroptosis-related lncRNAs may provide new insight into exploring the mechanisms of ferroptosis in GC cells and predicting GC patients 115-117. Furthermore, circRNAs are involved in GC progression and may become novel therapeutic targets for the prevention and treatment of GC 118. Chang Li 68 identified that circ_0008035 could promote GC cell proliferation and decrease iron accumulation and lipid peroxidation, resulting in the inhibition of cell ferroptosis via the miR-599/EIF4A1 axis in GC cells. This finding may contribute to the discovery of novel therapeutic targets for GC. Moreover, circRNAs act as regulators in the progression of CRC via ferroptosis 119. For instance, downregulation of circABCB10 via the miR-326/CCL5 axis promoted cancer cell ferroptosis and inhibited the progression of rectal cancer 71. Similarly, another study 70 showed that circ_0007142 was upregulated in CRC, and inhibition of circ_0007142 could promote apoptosis and ferroptosis of CRC cells, resulting in reduced cancer cell proliferation.

### 2.3 Liver cancer

In addition to gastrointestinal cancer, liver cancer is the third largest cause of cancer-triggered death, with approximately 8.3% in both sexes 99. The pathological types of liver cancer include hepatocellular carcinoma (HCC), cholangiocellular carcinoma, and mixed type. HCC is the most common pathological type of liver cancer. According to Barcelona Clinic Liver Cancer, the great majority of HCC patients are diagnosed after symptoms develop 120. If they miss the therapeutic window in the early stage, the disease will be very difficult to treat. Hence, it is necessary to study the pathogenesis of liver cancer more deeply, especially regarding moderate or advanced liver cancers, and explore novel therapeutic methods or accessible diagnostic biomarkers.

Several studies have focused on ferroptosis in liver cancer and explored its molecular mechanism in depth in recent years. Some studies have revealed the role of ncRNAs in the regulation of ferroptosis in HCC 69,74. Hence, Tao Bai 74 revealed that miR-214-3p (miR-214) could enhance erastin-induced ferroptosis by targeting ATF4 in HCC. In this research, overexpression of pre-miR-214 increased the levels of malondialdehyde (MDA), ROS, and Fe^2+^, and reduced the GSH levels when HepG2 and Hep3B cells were treated with erastin, whereas overexpression of anti-miR-214 demonstrated the opposite effect. In addition, some studies began to focus on the role of lncRNAs in the regulation of cellular ferroptosis in HCC. In 2019, Wenchuan Qi et al. 69 found that lncRNA GABPB1-AS1 could reduce GABPB1 protein levels by inhibiting GABPB1 translation during erastin-induced ferroptosis in HCC cells, resulting in the downregulation of PRDX5 protein. When PRDX5 localization in mitochondria to reduce peroxidases and hydroperoxides 121 is inhibited, the completeness of the cellular membrane and cell viability are destroyed. Moreover, it was revealed that lncRNA PVT1 played an important role in accelerating the expression of GPX4 and inhibiting ferroptosis by the miR-214-3p/GPX4 pathway 90. To identify biomarkers of prognostic prediction in HCC, many ferroptosis-related lncRNAs were validated and might become signatures for predicting the overall survival of HCC patients and novel therapeutic targets to affect HCC cell proliferation and invasion 122-125.

Similar to the roles of miRNAs and lncRNAs in liver cancer, circRNAs also demonstrate crucial regulatory roles in liver cancer cell ferroptosis. One study revealed that circIL4R, which is greatly upregulated in HCC tissues and cells, could inhibit ferroptosis by sponging miR-541-3p through the GPX4 pathway, resulting in the promotion of HCC tumorigenesis 91. Similarly, one report from Zhiqian Liu 126 demonstrated that circcIARS was abnormally overexpressed after sorafenib treatment and could promote sorafenib-induced ferroptosis in HCC cells by inhibiting RNA-binding protein ALKBH5-induced autophagy inhibition. Moreover, circRNAs can act as ceRNAs to regulate liver cell ferroptosis. Ning Lyu 84 identified circ0097009 as a ceRNA that sponged miR-1261 and upregulated the expression of SLC7A11, inhibiting HCC cell ferroptosis and promoting the invasion and metastasis of HCC cells.

Overall, ncRNAs may become potential therapeutic targets to treat HCC via the ferroptosis pathway.

### 2.4 Breast cancer

Breast cancer has exceeded lung cancer as the most frequently newly diagnosed cancer in both sexes and has the most cancer-related deaths among women worldwide. There were an estimated 2.3 million new cases (11.7%) in both sexes and 0.68 million deaths (15.5%) in females 99. There are many therapeutic methods, including surgery, chemotherapy, and radiotherapy, to treat breast cancer. Several studies have focused on the ferroptosis-related genes as biomarkers for diagnosing or treating and predicting prognosis in breast cancer 66. However, reducing the number of newly diagnosed cases and mortality due to breast cancer still presents huge challenges.

To improve the therapeutic effects of drugs, several studies have revealed that some drugs exhibit anticancer effects by regulating ncRNA expression to affect cancer cell ferroptosis 89. In this research, Yifeng Hou 89 discovered that metformin, a widely used antidiabetic drug, could lead to ferroptosis by upregulating miR-324-3p expression and downregulating GPX4 in breast cancer. This finding suggested that metformin could become a potential anticancer drug. Another study discovered that miR-5096 could increase the ROS, iron accumulation and lipid peroxidation by inhibiting SLC7A11 and inducing ferroptosis 127. In addition to miRNAs, some lncRNAs have been identified and play an important role in regulating the ferroptosis and cancer. For instance, Chao Mao 77 found that lncRNA P53RRA not only suppressed the progression of lung cancer, but also inhibited breast cancer growth by promoting cell ferroptosis. The exploration of breast cancer biomarkers for predicting prognosis might be helpful to improve therapeutic strategies via bioinformatic analysis. Many ferroptosis-related lncRNAs have been discovered and may become prognostic signatures or potential therapeutic targets for breast cancer 128,129. In addition, Huiming Zhang and his colleague 73 discovered that circRHOT1 could attenuate cancer cell ferroptosis through the miR-106a-5p/STAT3 pathway and promote the invasion and migration of breast cancer cells, leading to worsening progression of breast cancer. In HER-2-positive breast cancer, circGFRA1 could inhibit ferroptosis and promote cancer progression via the miR-1228/AIFM2 axis 130. These findings may provide new insight into exploring the regulatory mechanism. However, more studies are still needed to focus on the regulatory role of ncRNAs in breast cancer via ferroptosis.

### 2.5 Urogenital cancer

Urogenital cancer is also one of most common cancers, but its newly diagnosed cases and deaths are lower than those of female breast cancer. It consists of bladder cancer, renal cancer, ureteropelvic cancer and urinary tract cancer. Among these cancers, bladder cancer and renal cancer accounted for an estimated 1 million new cases (5.5%) and 0.37 million deaths (3.9%) worldwide 99. Although there are some therapeutic methods to treat it, exploring the novel diagnostic and therapeutic methods is still essential.

To explore novel diagnostic and therapeutic targets, accumulating evidence has focused on the role of ferroptosis-related ncRNAs in the genesis, progression, and treatment of urogenital cancer 131. In term of bladder cancer, lncRNA RP11-89 could induce tumorigenesis and reduce the accumulation of cellular iron by sponging the miR-129-5p/PROM2 pathway, and leading to ferroptosis inhibition 132. Moreover, several studies have revealed that some ferroptosis-associated lncRNAs could become prognostic signatures in renal clear cell carcinoma 133. More studies are needed to uncover more ferroptosis-related ncRNAs and explore their regulatory role of them in the urogenital cancer.

### 2.5 Prostate cancer

The number of new cases of prostate cancer is very high, next only to lung cancer in males, at an estimated 14.1%, but the cancer-related death rate is lower (approximately 4.5%) than that of lung cancer (approximately 21.5%) 99. The occurrence and progression of prostate cancer involve both genetic and environmental factors 134. In a recent study, Yangyi Zhang 135 demonstrated that chronic cadmium exposure could promote cancer cell growth and inhibit ferroptosis by upregulating lncRNA OIP5-AS1 expression, and lncRNA OIP5-AS1 acted as a ceRNA that sponges miR-128-3p to increase the level of SLC7A11. However, future studies are needed to reveal the regulatory role of ferroptosis-related ncRNAs in inhibiting the progression of prostate cancer.

### 2.6 Cervical cancer

Cervical cancer is the fourth most common cancer in women, with an estimated 6.5%, and ranks as the fourth leading cause of cancer-associated death in women, with approximately 7.7% 85,99. Although there are many therapeutic methods to treat it, such as chemotherapy and surgery, cervical cancer still lacks adequate and effective treatment to improve the low survival rate and poor prognosis. Recent studies have focused on the potential regulatory role of ncRNAs in improving the prognosis of cervical cancer via ferroptosis. For example, Peng Wu 85 showed that circEPSTI1 promoted the proliferation of cervical cancer via miR-375/409-3P/515-5p acting as a ceRNA by targeting SLC7A11 and attenuated the effect of lipid peroxidation and GSH/GSSG to inhibit ferroptosis of cervical cancer cells.

### 2.7 Ovarian cancer

Ovarian cancer is also a common gynecological cancer, with its number of deaths ranking eighth among gynecological cancers 99. The majority of ovarian cancers cannot be diagnosed at an early stage, and the 5-year survival rate is low. More studies should be conducted to discover the pathogenic mechanism of ovarian cancer. Currently, some evidence has revealed that ncRNAs play an important role in suppressing ovarian cancer via the ferroptosis pathway 136,137. Research revealed that miR-424-5p negatively regulated ferroptosis by directly targeting ACSL4, an overexpressed ferroptosis-related protein, in ovarian cancer cells and that downregulation of miR-424-5p increased erastin- and RSL3-induced ferroptosis, resulting in inhibition of the progression of ovarian cancer 97.

### 2.8 Acute myeloid leukemia

Acute leukemia (AL), including acute myeloid leukemia (AML) and acute lymphoblastic leukemia (ALL), is also a serious malignant disease 138. AL is a type of malignant clonal disease stemming from hematopoietic stem cells, and AML is very common in adults 138. The five-year survival rates of patients treated with chemotherapeutics were 27.4% 139 lower than those of patients treated with stem cell transplantations. Moreover, drug resistance is one of the major problems in chemotherapy for AML. Therefore, it is necessary to explore the mechanism of drug resistance to increase the therapeutic sensitivity of chemotherapy in AML. The abnormal expression of ncRNAs may be the key regulator to improve drug resistance 140. For example, Zuili Wang and his colleague 79 revealed that lncRNA LINC00618 was downregulated in human leukemia and strongly increased by VCR therapeutics, and it was involved in inducing AML cell ferroptosis by increasing the production of ROS and iron and decreasing the expression of SLC7A11 in AML.

However, ncRNAs, including miRNAs, lncRNAs and circRNAs, require further investigation regarding the chemotherapeutic resistance of AML.

### 2.9 Glioma

Although less common in other cancers, glioma is the most common primary central nervous system tumor and accounts for approximately half of all primary intracranial tumors. The five-year survival rate of adult high-grade glioma is very low. According to the WHO grade, low-grade glioma is grade I or II, and high-grade glioma is grade III or IV among the four grades of glioma 141. Grade IV glioma is also named glioblastoma (GBM). Hence, it is essential to explore the regulatory mechanism of glioma and some novel therapeutic methods. Recently, some studies have reported that some ncRNAs play important roles in inhibiting glioma via ferroptosis 76,98. Xinzhi Yang 98 revealed that miR-18a accelerated glioblastoma advancement by directly inhibiting ALOXE3-mediated ferroptotic and antimigration activities. Deficiency of ALOXE3 in GBM cells results in resistance to p53-SLC7A11-associated ferroptosis and improves the survival rate of GBM cells. Moreover, it was reported that the circRNA TTBK2 was upregulated in glioma tissues and cells and inhibited ferroptosis via the miR-761/ITGB8 axis to promote glioma proliferation and invasion 76.

### 2.10 Head and neck squamous cell carcinoma

Head and neck squamous cell carcinoma (HNSCC) is a series of malignant tumors involving many tissues in the head and neck region, including the oral cavity, nasopharynx and throat 142. Although surgery, radiotherapy and chemotherapy are available, the morbidity of HNSCC has increased markedly in recent years, especially in women 99. It is particularly important to explore the regulatory mechanism and uncover novel remarkable biomarkers and therapeutic targets to improve the progression of HNSCC. Bin Zhang revealed that miR-125b-5p could inhibit the expression of SLC7A11 and that enhancer of zeste homolog 2 (EZH2) inhibited ferroptosis via the miR-125b-5p/SLC7A11 pathway in tongue squamous cell carcinoma 87. Moreover, Yun Tang 143 identified that some ferroptosis-related lncRNAs may become diagnostic biomarkers and potential therapeutic targets to treat HNSCC via ferroptosis. Furthermore, some research has focused on the role of ferroptosis-related circRNAs in HNSCC. For example, circFNDC3B could inhibit cancer cell ferroptosis by miR-520d-5p/SLC7A11 pathway in oral squamous cell carcinoma 78. Another study revealed that circKIF4A could upregulate the levels of GPX4 and reduce the ferroptosis of thyroid cancer cells by sponging miR-1231, and leading to the induction of cancer progression 92. Moreover, circ_0067934 reduced lipid peroxidation and ferroptosis of thyroid cancer cells via the miR-545-3p/SLC7A11 pathway 144. However, more evidence is needed to identify the regulatory roles of these ncRNAs in HNSCC via ferroptosis.

### 2.11 Melanoma

In contrast to other cancers, melanoma stemming from melanocytes is not common. However, melanoma is the third most frequently occurring malignant tumor of the skin. Melanoma lacks specific treatment, except for surgical resection in the early stage. Hence, ferroptosis is a focus of researchers, and new cell death contributes to inhibiting melanoma progression. Meiying Luo 81 revealed that miR-137 could inhibit glutamine transporter SLC1A5, an inhibitor of ferroptosis, in melanoma cells and that the suppression of SLC1A5 decreased glutamine uptake and MDA accumulation, resulting in ferroptosis and inhibiting the progression of melanoma. In another study, Kexin Zhang 95 identified that overexpression of miR-9 inhibited GOT1, leading to reduced erastin- and RSL3-mediated ferroptosis. Suppression of miR-9 could increase the levels of lipid ROS in melanoma cells, leading to promotion of ferroptosis in melanoma cells and the inhibition of melanoma growth 95. However, other ncRNAs, including lncRNAs and circRNAs, ought to be focused on to define the regulatory role of ferroptosis in melanoma.

### 2.12 Clinically relevant radioresistance

Like chemotherapy, radiation therapy (RT) is also one of the most common therapies for cancers. However, radioresistance decreases the therapeutic effect of RT, and the mechanism of radioresistance is not well understood. In recent years, Kazuo Tomita 83 revealed that miR-7-5p played a crucial role in regulating irradiation resistance by controlling intracellular Fe^2+^ content in clinically relevant radioresistant (CRR) cells. Oxidative stress and ferroptosis in CRR cells are inhibited. In the future, more investigations will be required to uncover the mechanism by which miRNAs improve radioresistance in cancer cells via the ferroptosis pathway.

## 3. Conclusions and future prospects

Ferroptosis, a newly discovered form of programmed cell death, is related to some pathophysiological processes, especially many types of cancers. Numerous studies have focused on exploring the regulatory mechanisms of cancers via the ferroptosis pathway. Great progress in exploring the regulatory role of ncRNAs in cancers by ferroptosis has been made. Taken together, these findings contribute to further understanding the pathogenesis of cancers and have demonstrated that ferroptosis-associated ncRNAs may act as a series of tumor inhibitors to suppress cancer growth. In addition, ncRNAs, including miRNAs, lncRNAs and circRNAs, have the potential to be novel anticancer therapeutic methods and diagnostic biomarkers by regulating the ferroptosis of cancer cells (Table 2).

Many studies have revealed that these ncRNAs play important roles in the progression of cancers via ferroptosis and that these ncRNAs may regulate the ferroptosis of cancer cells to induce or inhibit tumorigenesis. It has been demonstrated that lncRNAs and circRNAs always sponge miRNAs to regulate the expression of GPX4 and induce lipid peroxidation of the cellular membrane. Furthermore, abnormal lipid peroxidation destroys cancer cell membranes, resulting in the ferroptosis of cancer cells. Sometimes, these ncRNAs can directly target GPX4, SLC3A2 and SLC7A11 to induce ferroptosis in cancer cells. In addition, some ncRNAs regulate the ferroptosis of cancer cells via the lethal accumulation of ROS and the abnormal metabolism of iron.

Due to the individual heterogeneity of ncRNA expression in different types of cancer, ncRNA-associated therapy and biomarkers may be applied to support personalized cancer treatment. Although an increasing number of studies have revealed a regulatory mechanism between cancer and ferroptosis, a deeper understanding of the mechanism by which ferroptosis-related ncRNAs regulate the progression and growth of cancer is still needed. Moreover, future studies should pay more attention to the role of ncRNAs in the linkage between cancer and ferroptosis. Moreover, some biomaterials, like nanomaterials, may overcome the shortcomings of conventional therapeutic schedules for tumor-targeted ferroptosis therapy by preloading antitumor drugs 145,146. However, the regulatory relationship between these promising materials and ferroptosis-related ncRNAs still lacks of research and should be given more attention.

This review has summarized the regulatory roles of several types of ncRNAs in cancer progression and ferroptosis. These studies are beneficial for understanding the pathogenesis of cancer. Ferroptosis-related ncRNAs have great potential to act as anticancer therapeutic targets by regulating ferroptosis. Targeting these key ncRNAs may reveal novel therapeutic methods or diagnostic biomarkers to inhibit the growth and progression of malignant tumors.

## Figures and Tables

**Table 1:** The regulatory role of ncRNAs in cancer progression via ferroptosis of cancer cells.

Cancers	NcRNAs	Mechanism	Function	Reference
Lung cancer	exo-miR-4443	Target METTL3/FSP1	Induce ferroptosis and increase cisplatin resistance of NSCLC	67
	miR-324-3p	Target GPX	Induce ferroptosis and reduce cisplatin resistance	88
	miR-27a-3p	Target SLC7A11	Induce ferroptosis and inhibit lung cancer	86
	lncRNA P53RRA	Interact with G3BP1	Induce ferroptosis and inhibit lung cancer	77
	LINC00336	Sponge with miR-6825 as ceRNA	Inhibit ferroptosis and promoting lung cancer	82
	lncRNA mir503HG	Sponge with miR-1273c as ceRNA	Induce ferroptosis and inhibit NCSLC	103
	lncRNA NEAT1	Upregulate the expression of ACSL4	Induce ferroptosis and inhibit NCSLC	96
	lncRNA MT1DP	target miR-365a-3p/NRF2	Induce ferroptosis and inhibit NCSLC	72
	lncRNA ASMTL-AS1	Stabilzing SAT1 and recruiting U2AF2	Induce ferroptosis and inhibit NCSLC	104
	circDTL	Target miR-1287-5p/GPX4	Inhibit ferroptosis and promote lung cancer	108
Gastrointestinal cancer	miR-4715-3p	DNA methylation and downregulate AURKA	Induce ferroptosis and inhibit UGC	75
	exo-miR-522	Interact with ALOX15	Inhibit ferroptosis and promote chemotherapeutic resistance of GC	80
	miR-103a-3p	Target GLS2	Induce ferroptosis and inhibit GC	94
	miR-139-5p	Target SLC7A11	Induce ferroptosis and inhibit pancreatic carcinoma	109
	miR-375	Target SLC7A11	Induce ferroptosis and inhibit GC	110
	circ_0008035	Target miR-599/EIF4A1	Inhibit ferroptosis and promote GC	68
	circABCB10	Target miR-326/CCL5	Induce ferroptosis and inhibit rectal cancer	71
	circ_0007142	Target miR-874-3p/GDPD5	Induce ferroptosis and inhibit CRC	70
Liver cancer	miR-214-3p	Target ATF4	Induce ferroptosis and inhibit HCC	74
	lncRNA GABPB1-AS1	Downregulation of GABPB1 and PRDX5	Induce ferroptosis and inhibit HCC	69
	lncRNA PVT1	Target miR-214-3p/GPX4	Inhibit ferroptosis and promote liver cancer	90
	circIL4R	Target miR-541-3p/GPX4	Inhibit ferroptosis and promote HCC	91
	circcIARS	Target ALKBH5	Induce ferroptosis and inhibit HCC after SF treatment	126
	circ_0097009	Target miR-1261/SLC7A11	Inhibit ferroptosis and promote HCC	84
Breast cancer	miR-324-3p	Target GPX4	Induce ferroptosis and inhibit breast cancer	89
	miR-5096	Target SLC7A11	Induce ferroptosis and inhibit breast cancer	127
	lncRNA P53RRA	Interact with G3BP1	Induce ferroptosis and inhibit breast cancer	77
	circGFRA1	Target miR-1228/AIFM2	Inhibit ferroptosis and promote breast cancer	130
	circRHOT1	Target miR-106a-5p/STAT3	Inhibit ferroptosis and promote breast cancer	73
Urogenital cancer	lncRNA RP11-89	Target miR-129-5p/PROM2	Inhibit ferroptosis and promote urogenital cancer	132
Prostate cancer	lncRNA OIP5-AS1	Target miR-128-3p/SLC7A11	Inhibit ferroptosis and promote prostate cancer	135
Cervical cancer	circEPSTI1	Target miR-375/409-3P/515-5p/SLC7A11	Inhibit ferroptosis and promote cervical cancer	85
Ovarian cancer	miR-424-5p	Target ACSL4	Inhibit ferroptosis and promote ovarian cancer	97
Acute myeloid leukemia	LINC00618	Downregulate SLC7A11	Induce ferroptosis and inhibit Acute myeloid leukemia	79
Glioma	miR-18a	Downregulate ALOXE3	Inhibit ferroptosis and promote glioma	98
	circTTBK2	Target miR-761/ITGB8	Inhibit ferroptosis and promote glioma	76
Head and neck squamous cell carcinoma	miR-125b-5p	Target SLC7A11	Inhibit ferroptosis and promote tongue squamous cell carcinoma	87
	circFNDC3B	Target miR-520d-5p/SLC7A11	Inhibit ferroptosis and promote orall squamous cell carcinoma	78
	circKIF4A	Target miR-1231/GPX4	Inhibit ferroptosis and promote papillary thyroid cancer	92
	circ_0067934	Target miR-545-3p/SLC7A11	Inhibit ferroptosis and promote thyroid cancer	144
Melanoma	miR-137	Downregulate SLC1A5	Induce ferroptosis and inhibit melanoma	81
	miR-9	Downregulate GOT1	inhibit ferroptosis and promote melanoma	95
Clinically relevant radioresistance	miR-7-5p	-	Inhibit ferroptosis and promote CRR	83

**Table 2:** The therapeutic target and diagnostic or prognostic biomarker of cancers.

Cancers	Therapeutic Target	Diagnostic or Prognostic Biomarker
Lung cancer	exo-miR-4443 67	miR-27a-3p 86
	miR-324-3p 88	lncRNA C20orf197 105
	miR-27a-3p 86	lncRNA ARHGEF26-AS1 105
	LINC00336 82	lncRNA MGC32805 105
	lncRNA NEAT1 96	lncRNA LINC00324 105
	lncRNA MT1DP 72	lncRNA LINC01116 105
	circDTL 108	lncRNA LINC01137 105
	-	lncRNA TMPO-AS1 105
	-	lncRNA AC021016.1 106
	-	lncRNA AC068228.2 106
	-	lncRNA MIR223HG 106
	-	lncRNA AC009275.1 106
	-	lncRNA AL049555.1 106
	-	lncRNA KTN1-AS1 106
Gastrointestinal cancer	exo-miR-522 80	miR-375 110
	miR-103a-3p 94	LINC01503 114
	miR-139-5p 109	lncRNA AC004687.1 114
	circ_0008035 68	lncRNA AC010973.2 114
	circABCB10 71	lncRNA AP001189.3 114
	circ_0007142 70	lncRNA ARRDC1-AS1 114
	-	lncRNA OIP5-AS1 114
	-	lncRNA NCK1-DT 114
	-	-
Liver cancer	miR-214-3p 74	lncRNA RHPN1-AS1 122
	lncRNA GABPB1-AS1 69	lncRNA MAPKAPK5-AS1 122
	circIL4R 91	lncRNA PART1 122
	circ_0097009 84	LINC00324 123
	-	lncRNA MSC-AS1 123
	-	lncRNA AC023157.3 123
	-	lncRNA AC0090005.1 123
	-	lncRNA PRRT3-AS1 123
	-	lncRNA AC015908.3 123
	-	lncRNA AC145207.5 123
	-	lncRNA AL031985.3 123
	-	lncRNA TMEM220-AS1 123
	-	lncRNA LUCAT1 124
	-	lncRNA AC099850.3 124
	-	lncRNA AL365203.2 124
	-	lncRNA AL031985.3 124
	-	lncRNA CTD-2033A16.3 125
	-	lncRNA CTD-2116N20.1 125
	-	lncRNA CTD-2510F5.4 125
	-	lncRNA DDX11-AS1 125
	-	lncRNA ZFPM2-AS1 125
	-	LINC00942 125
	-	LINC01224 125
	-	LINC01231 125
	-	LINC01508 125
	-	circ_0097009 84
Breast cancer	miR-5096 127	lncRNA AC10874.1 128
	circRHOT1 73	lncRNA AL133467.1 128
	circGFRA1 130	LINC01235 128
	-	lncRNA AC072039.2 128
	-	lncRNA TDRKH-AS1 128
	-	lncRNA USP30-AS1 128
	-	lncRNA MAPT-AS1 128
	-	lncRNA LIPE-AS 128
	-	lncRNA AC121247.2 129
	-	lncRNA LIPE-AS1 129
	-	lncRNA HSD11B1-AS1 129
	-	lncRNA AC010655.2 129
	-	lncRNA PTPRD-AS1 129
	-	lncRNA AC099329.2 129
	-	lncRNA OTUD6B 129
	-	LINC01871 129
	-	LINC00393 129
	-	LINC02384 129
	-	LINC01419 129
	-	LINC02266 129
Urogenital cancer	lncRNA RP11-89 132	lncRNA DUXAP8 133
	-	lncRNA LUCAT1 133
	-	LINC02609 133
Prostate cancer	lncRNA OIP5-AS1 135	-
Cervical cancer	circEPSTI1 85	circEPSTI1 85
Ovarian cancer	miR-424-5p 97	-
Glioma	miR-18a 98	miR-18a 98
	circTTBK2 76	-
Head and neck squamous cell carcinoma	miR-520d-5p 78	LINC01963 143
	miR-125b-5p 87	
	LINC01963 143	LINC01980 143
	LINC01980 143	lncRNA AATBC 143
	lncRNA AATBC 143	lncRNA ELF3-AS1 143
	lncRNA ELF3-AS 143	-
	circFNDC3B 78	-
	circKIF4A 92	-
	circ_0067934 144	-
Melanoma	miR-137 81	-
	miR-9 95	-
Clinically relevant radioresistance	miR-7-5p 83	-

## Abbreviations

ncRNAs: noncoding RNAs
PCD: programmed cell death
TFRC: transferrin receptor
SLC40A1: solute carrier family 40 membrane 1
NUPR1: nuclear protein 1
GPX4: glutathione peroxidase 4
Glu: glutamate
Cys_2_: cysteine
SLC3A2: solute carrier family 3 membrane 2
SLC7A11: solute carrier family 7 membrane 11
BAP1: BRCA1-related protein
KEAP1: Kelch-like ECH-associtaed protein 1
NRF2: NF E2-related factor 2
PGRMC1: progesterone receptor membrane component 1
mTORC1: rapamycin complex 1
AA: arachidonic acid
ACSL4: acyl-CoA synthetase long-chain family member 4
LPCAT3: lysophophatidylcholne acyltransferase 3
LOXs: lipoxygenases
miRNAs: microRNAs
lncRNAs: long noncoding RNAs
circRNAs: circular RNAs
GLS2: glutaminase 2
GOT1: glutamic-oxaloacetic transaminase 1
SLC1A5: solute carrier family 1 membrane 5
SCLC: small cell lung cancer
NSCLC: non-small cell lung cancer
ADC: adenocarcinoma
SCC: squamous cell carcinoma
ceRNA: competing endogenous RNA
UGCs: upper gastric cancers
CRC: colorectal cancer
HCC: hepatocellular carcinoma
MDA: malondialdehyde
AL: Acute leukemia
AML: acute myeloid leukemia
ALL: acute lymphoblastic leukemia
GBM: glioblastoma
HNSCC: head and neck squamous cell carcinoma
EZH2: enhancer of zeste homolog 2
RT: radiation therapy
CRR: clinically relevant radioresistant
